# Probing Membrane Association of α-Synuclein Domains with VDAC Nanopore Reveals Unexpected Binding Pattern

**DOI:** 10.1038/s41598-019-40979-8

**Published:** 2019-03-14

**Authors:** Daniel Jacobs, David P. Hoogerheide, Amandine Rovini, Zhiping Jiang, Jennifer C. Lee, Tatiana K. Rostovtseva, Sergey M. Bezrukov

**Affiliations:** 1 Section on Molecular Transport, Eunice Kennedy Shriver National Institute of Child Health and Human Development, National Institutes of Health, Bethesda, MD 20892 USA; 2 Center for Neutron Research, National Institute of Standards and Technology, Gaithersburg, MD 20899 USA; 3 Laboratory of Protein Conformation and Dynamics, Biochemistry and Biophysics Center, National Heart, Lung, and Blood Institute, National Institutes of Health, Bethesda, MD 20892 USA

## Abstract

It is well established that α-synuclein (α-syn) binding from solution to the surface of membranes composed of negatively charged and/or non-lamellar lipids can be characterized by equilibrium dissociation constants of tens of micromolar. Previously, we have found that a naturally occurring nanopore of the mitochondrial voltage-dependent anion channel (VDAC), reconstituted into planar bilayers of a plant-derived lipid, responds to α-syn at nanomolar solution concentrations. Here, using lipid mixtures that mimic the composition of mitochondrial outer membranes, we show that *functionally important* binding does indeed take place in the nanomolar range. We demonstrate that the voltage-dependent rate at which a membrane-embedded VDAC nanopore captures α-syn is a strong function of membrane composition. Comparison of the nanopore results with those obtained by the bilayer overtone analysis of membrane binding demonstrates a pronounced correlation between the two datasets. The stronger the binding, the larger the on-rate, but with some notable exceptions. This leads to a tentative model of α-syn-membrane interactions, which assigns different lipid-dependent roles to the N- and C-terminal domains of α-syn accounting for both electrostatic and hydrophobic effects. As a result, the rate of α-syn capture by the nanopore is not simply proportional to the α-syn concentration on the membrane surface but found to be sensitive to the specific interactions of each domain with the membrane and nanopore.

## Introduction

The exceptional diversity of interactions between peripheral membrane proteins and bilayer lipid membranes makes the membrane surface a rich scene for performing and regulating cellular functions^1^. This same diversity presents significant experimental barriers to studies of interaction mechanisms among the many participating components. One of the main challenges is that the energies of interaction between individual protein residues and lipid molecules are small; thus, statistical effects play a significant role. A variety of techniques to characterize binding of peripheral membrane proteins to liposome or planar lipid bilayer platforms have been developed, but it is increasingly clear that, except in the simplest of systems, no isolated technique yields a clear picture of the membrane binding process. In part, this is because these assays measure the average binding parameters of an ensemble of molecules, highlighting the need to characterize membrane-bound proteins at the single-molecule level. In this report, we use the voltage-dependent anion channel (VDAC)—a bilayer-embedded, naturally occurring nanopore—as a single-molecule probe for α-synuclein (α-syn), a peripheral membrane protein of considerable clinical interest. We show that significant membrane binding of α-syn takes place at concentrations that are three orders of magnitude smaller than the equilibrium dissociation constants deduced from most macroscopic measurements. Crucially, this binding is functionally important, *i*.*e*. able to modulate metabolite (such as ATP and ADP) fluxes through VDAC, for the lipid compositions that closely mimic those of the mitochondrial outer membrane (MOM).

α-Syn is a small, intrinsically disordered cytoplasmic protein that is highly expressed in the central nervous system and is involved in the pathology of Parkinson disease (PD)^2^. α-Syn is the major component of the Lewy bodies in the brain of PD patients^3^, but the precise physiological role of monomeric α-syn or Lewy bodies remains unclear. Although α-syn is primarily localized in the cytosol, its association with the variety of cellular membranes is now well established^4,5^. An impressive number of detailed *in vitro* investigations have already been devoted to understanding the mechanism of α-syn binding to lipids (see e.g.^6–9^). It was shown that while α-syn is disordered in bulk solutions, it adopts a secondary structure upon binding to lipid membranes^8–12^. In addition to interacting with synaptic vesicle membranes, α-syn was also found to specifically bind to the native mitochondrial membranes in live cells^13–16^. Recent studies demonstrate α-syn involvement in mitochondrial dysfunction in neurodegeneration and neuroapoptosis^17–20^. Therefore, understanding the molecular mechanism of α-syn interaction with model membranes mimicking mitochondrial membranes is of particular importance.

There are three distinctive amino acid regions of α-syn: the weakly positively charged N-terminal domain, the central nonpolar domain, and the highly acidic C-terminal domain containing 15 negative charges^8^. The N-terminal domain of α-syn has been unambiguously identified as its lipid-binding domain which acquires helical structures upon association with different lipid membrane surfaces^8,10,12,21^. The presence of negatively charged lipids is generally believed to be essential for α-syn membrane binding^21^, which is attributed to the electrostatic attraction of multiple lysines in the N-terminal region. However, even in high ionic strength solutions the helical conformation of membrane-bound α-syn is preserved, pointing to the involvement of hydrophobic interactions^8,21^. While α-syn prefers anionic lipids, it also has considerable binding affinity to zwitterionic lipids^22^, with a preference to nonlamellar phosphatidylethanolamine (PE)^23^. In addition to having a strong curvature sensitivity^24^, α-syn affects the morphology of membrane surfaces to which it binds^25–27^. In summary, lipids with small and/or negatively charged headgroups enhance both binding affinity and helix formation of α-syn.

VDAC is a β-barrel channel characterized by weak anion selectivity and gating under applied voltage^28,29^. It is a mitochondrial outer membrane protein, which constitutes a major pathway for water-soluble metabolites in and out of mitochondria^28–32^. Due to its large inner diameter of ~2.7 nm^33^, in addition to the conventional mitochondrial anionic substrates such as ATP and ADP, the VDAC nanopore suits nicely to translocate disordered polypeptide chains^34,35^, which are preferably negatively charged to fit its anionic selectivity, accounted for by the net positive charge of 3^33–35^. Recently it was found that VDAC reconstituted into planar lipid membranes, made of  the plant lipid diphytanoylphosphatidylcholine, can be reversibly blocked by α-syn with nanomolar efficiency^36^. Based on the detailed kinetic analysis of the blocking events, a hypothetical model for α-syn interaction with the VDAC nanopore was proposed. According to this model, the capture of the negatively charged C-terminal domain of α-syn by the anionic VDAC nanopore, revealed through the characteristic transient ionic current blockages, is necessarily preceded by α-syn binding to the membrane surface^36^.

Previously published studies that reported effects of lipid headgroups, acyl chain structure, membrane surface tension, and curvature on α-syn membrane binding have been mainly focused on α-syn-induced membrane deformation such as area expansion or tubulation^25,26^. The characteristic dissociation constants were measured to range from 2 to 2000 μM (M = mol/l) depending on the liposome lipid composition and membrane curvature^24,27,37^. Instead, the present work focuses on the molecular features of the recently discovered α-syn binding in the nanomolar range. We are interested in how lipid composition changes the strength of the binding and influences α-syn interaction with VDAC nanopore. We show that this interaction is strongly affected by changes in the membrane lipid composition. We focus on the rate at which α-syn molecules block the voltage-biased nanopore ionic current (the “on-rate”). These single-molecule results are compared with macroscopic measurements of α-syn binding to planar bilayer architectures using the bilayer overtone analysis (BOA). We find that in 150 mM KCl the dependences of α-syn binding efficiency on the charge state of the lipids are different when probed by the nanopore versus the macroscopic BOA measurements, allowing us to hypothesize that α-syn can adopt multiple sub-conformations on membrane surfaces and that different sub-conformations vary in their propensity to be captured by the nanopore. The VDAC nanopore thus proves to be an extremely sensitive single-molecule probe for peripheral membrane binding.

## Results

### The rate of VDAC blockage by α-syn strongly depends on membrane lipid composition

Two zwitterionic lipids, dioleoylphosphatidylcholine (DOPC) and dioleoylphosphatidylethanolamine (DOPE), were chosen in our study to mimic MOM composition where PC and PE make up 44 and 35% of the total lipid content of the rat liver MOM, respectively^38^. To model the relatively high content of the negatively charged lipids in the MOM (up to 20%), negatively charged dioleoylphosphatidylglycerol (DOPG) and cardiolipin (CL) were used. The positively charged synthetic lipid dioleoyl-trimethylammonium-propane (DOTAP) was chosen as an antipode of DOPG to study the effect of lipid charge in more detail. The acyl chain composition was kept constant for all lipids (except for CL) to focus on the effect of lipid headgroups on α-syn binding.

**Figure 1:**
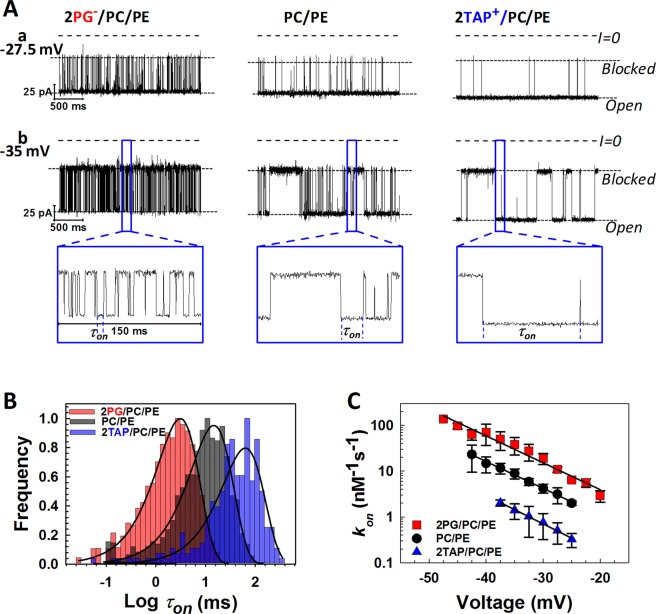
VDAC blockage by α-syn strongly depends on membrane lipid composition. (**A**) Representative current records of VDAC single-channels reconstituted into planar membranes formed from mixtures of 2PG/PC/PE (*left traces*), PC/PE (*middle traces*), and 2TAP/PC/PE (*right traces*) in 1 M KCl in the presence of 10 nM of α-syn added to the *cis* compartment. Traces were taken at −27.5 mV (*traces a*) and at −35 mV (*traces b*) of applied voltages. Insets in (*b*) show fragments of current records specified by boxes at finer time scale. Here and in Fig. 3A horizontal dotted lines indicate VDAC open and blocked states; dashed lines indicate zero current. Current traces were digitally filtered using a 5 kHz lowpass Bessel filter. The membrane-bathing solution contained 1 M KCl buffered by 5 mM HEPES at pH 7.4. (**B**) The log-binned distributions of the open time *τ*_*on*_ at −35 mV obtained in membranes formed from 2PG/PC/PE (*red*), PC/PE (*black*), and 2TAP/PC/PE (*blue*) lipid mixtures and acquired from statistical analysis of the current records as in (**A**). Solid lines are logarithmic single exponential fittings with characteristic times 〈*τ*_*on*_〉 equal to 3.1, 14.8, and 63.4 ms for 2PG/PC/PE, PC/PE, and 2TAP/PC/PE membranes, respectively. (**C**) Voltage dependences of the on-rate constant *k*_*on*_ of α-syn-VDAC binding in the membranes of different lipid compositions indicated by color code. The solid lines represent a fit to *k*_*on*_(*V*) = *k*_0_ exp (*n*_*g*_*e*|*V*|/(*k*_*B*_*T*)), where *V* is the applied voltage, *n*_*g*_ is the effective “gating charge” of 3.7 ± 0.5, and *e*, *k*_*B*_, and *T* have their usual meaning of the elementary charge, Boltzmann constant, and absolute temperature, respectively. Data points represent the mean of at least three independent experiments ± S. D. (*error bars*).

The current recordings from representative experiments shown in Fig. 1A demonstrate a significant difference in the frequency of blockage events induced by 10 nM of α-syn added to the both sides of the membranes formed from DOPG/DOPC/DOPE (2:1:1, mol/mol) (2PG/PC/PE) (*left traces*), DOPC/DOPE (1:1, mol/mol) (PC/PE) (*middle traces*), and DOTAP/DOPC/DOPE (2:1:1, mol/mol) (2TAP/PC/PE) (*right traces*) in 1 M KCl at −27.5 mV (*traces a*) and −35 mV (*traces b*) applied voltages. Measurable blockage of the VDAC nanopore occurs when negative potential is applied from the side of α-syn addition^36^. Although α-syn blocks VDAC in a qualitatively similar way from both sides of the channel^36^, here, for the sake of clarity, we present results for the blockage from the *cis* side only, the side of VDAC addition and apparently the cytosolic side of the channel^39^. Considering α-syn’s cytosolic localization in cells, its interaction with the cytosolic side of the channel is most physiologically relevant.

**Figure 2:**
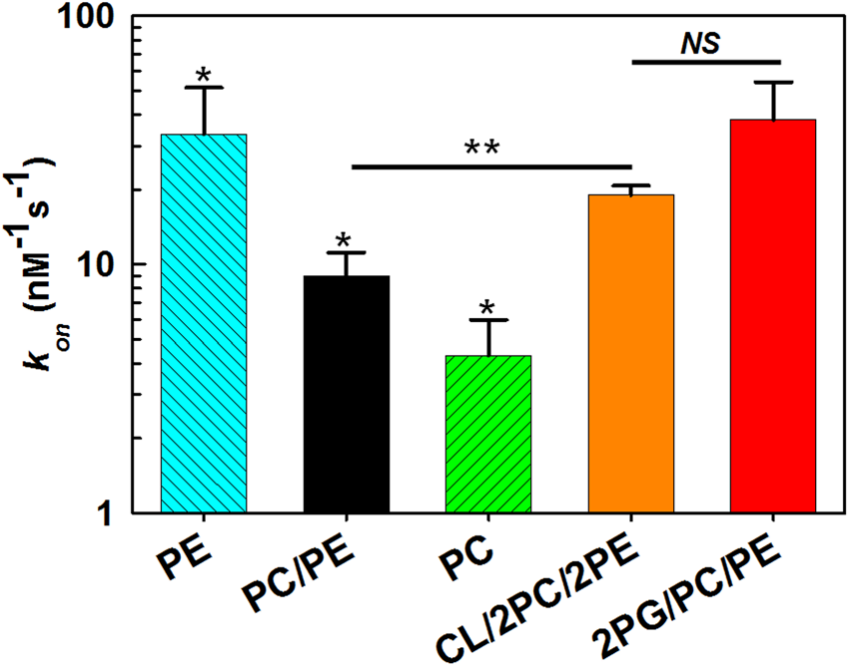
The on-rate of α-syn-VDAC increases in the presence of nonlamellar lipids. *k*_*on*_ were obtained in PE, PC/PE, PC, CL/2PC/2PE, and 2PG/PC/PE membranes in 1 M KCl buffer solutions at −35 mV of applied voltage. Significance between PE, PC/PE, and PC membranes was determined using one-way ANOVA (*p < 0.01). The significance of the effect of CL addition on *k*_*on*_ between CL/2PC/2PE and PC/PE membranes was determined using a two-tailed *t*-test (**p < 0.001). There is no significant difference of *k*_*on*_ values between CL/2PC/2PE, and 2PG/PC/PE membranes (NS: not significant, *p* > 0.05). Data are mean of at least three independent experiments ± S. D. (*error bars*).

The highest frequency of blockages is observed in the negatively charged membranes with 50% PG content (*left traces*, Fig. 1A) and the lowest frequency in the positively charged membranes with 50% TAP in the PC/PE mixture (*right traces*). In the neutral PC/PE membranes, α-syn blocks VDAC with frequencies between those for the negatively and positively charged membranes (*middle traces*). The difference in blockage efficiency can be seen more clearly at a finer time scale (insets in Fig. 1A,b), where the open time of the channel, *τ*_*on*_, i. e., the time when channel is open between the consequent blockages, increases from negative to neutral to positively charged membranes. For quantification of the blockage efficiency, the distributions of *τ*_*on*_ were analyzed for all membrane compositions and applied potentials. Figure 1B shows that at the −35 mV applied potential the characteristic open time, 〈*τ*_*on*_〉, corresponding to the average of the *τ*_*on*_ distributions, increases from 3.1 ms in 2PG/PC/PE to 14.8 and 63.4 ms in PC/PE and 2TAP/PC/PE membranes, respectively. The on-rate constant of the blockage, *k*_*on*_ = 1/(〈*τ*_*on*_〉 [*C*]), where [*C*] is α-syn bulk concentration, is an exponential function of the applied voltage^36^ with the similar slopes for all studied lipid compositions, as is illustrated in Fig. 1C. The blockage frequency increases by more than 30 times in anionic membranes compared with cationic ones. Interestingly, it is not only the lipid headgroup charge but also the type of the neutral headgroup that affects α-syn-VDAC binding. In 1 M KCl buffer solutions, there is almost 10 times difference in *k*_*on*_ between pure DOPE (PE) and DOPC (PC) membranes (Fig. 2), with *k*_*on*_ increasing with PE/PC ratio.

CL is a signature lipid of the mitochondrial inner membrane, which distribution in the MOM increases upon some stress conditions such as apoptosis^40^. We observe that the presence of 20 mol% of doubly negatively charged CL in the PC/PE mixture causes ~2 times increase of *k*_*on*_ compared with PC/PE membranes (Fig. 2). In the absence of a specific α-syn-CL interaction, one would expect the increase in on-rates of α-syn bound to PC/PE mixtures with 20 mol% doubly charged CL to be similar to the increase in on-rates of α-syn bound to mixtures with about 40 mol% of a singly charged lipid. Indeed, we find no significant difference between *k*_*on*_ obtained in CL/2PC/2PE and 2PG/PC/PE membranes (analyzed by two-tailed *t*-test) (Fig. 2). The increase of *k*_*on*_ is approximately a factor of 4. A specific α-syn-CL interaction, on the other hand, would be expected to manifest itself by a much larger increase than that induced by the presence of singly charged lipids accounting for a similar surface charge density. This suggests that the α-syn-VDAC binding kinetics do not detect the earlier proposed specificity of α-syn-CL binding^37^ but merely report on the effect of the CL headgroup negative charge and nonlamellar features on VDAC blockage.

**Figure 3:**
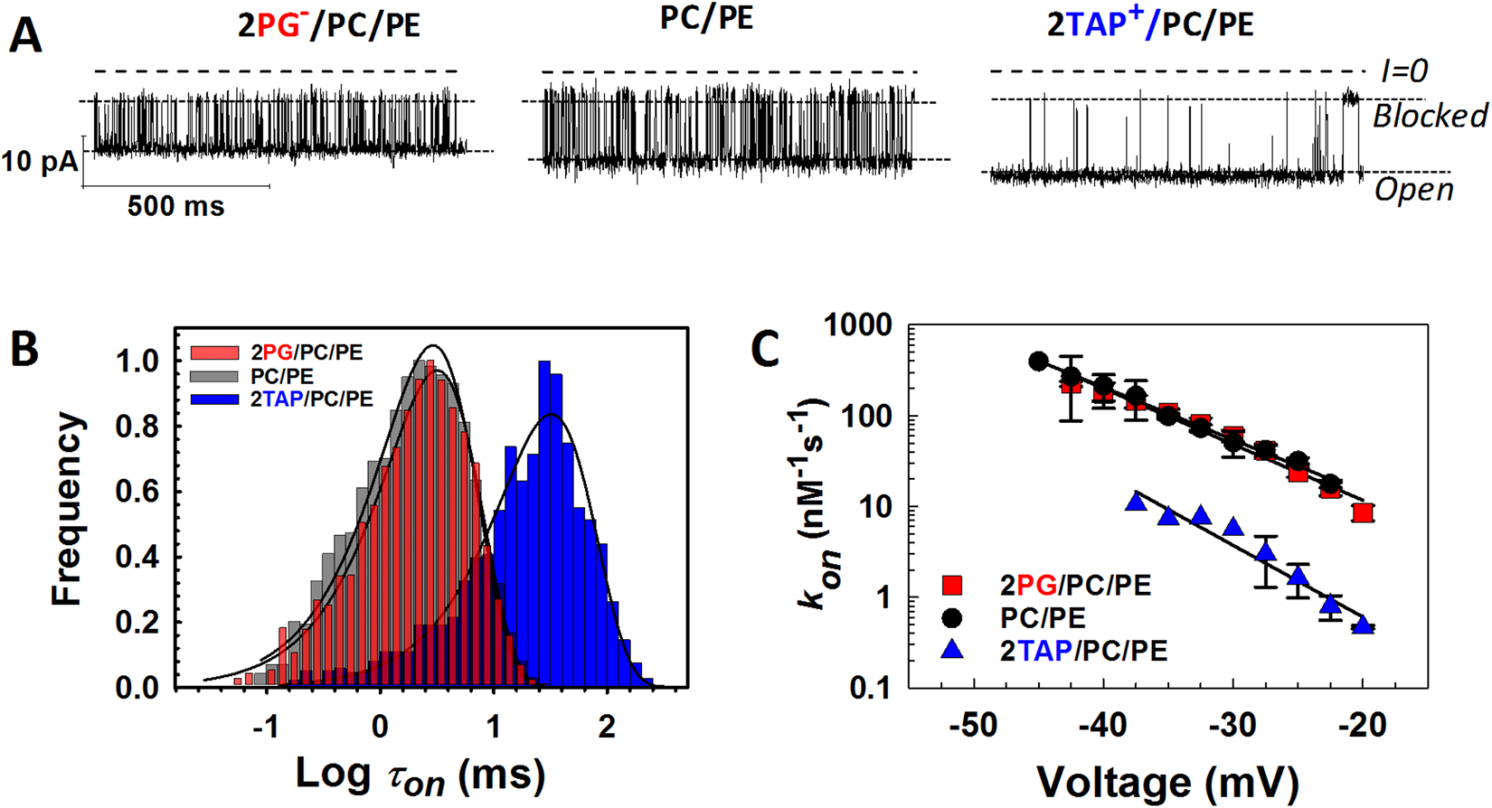
Kinetics of VDAC blockage by α-syn in the membranes of different lipid compositions in physiologically low salt. (**A**) Representative current records of VDAC single-channels reconstituted into planar membranes formed of 2PG/PC/PE (*left traces*), PC/PE (*middle traces*), and 2TAP/PC/PE (*right traces*) in 150 mM KCl in the presence of 10 nM of α-syn added to the *cis* compartment. Current traces were taken at −27.5 mV and digitally filtered using a 2 kHz lowpass Bessel filter. (**B**) The log-binned distributions of the open time *τ*_*on*_ at −27.5 mV obtained in membranes formed from 2PG/PC/PE (*red*), PC/PE (*black*), and 2TAP/PC/PE (*blue*) lipid mixtures and acquired from statistical analysis of the current records as in (**A**). Solid lines are logarithmic single exponential fittings with characteristic times 〈*τ*_*on*_〉 equal to 3.2, 2.7, and 32 ms for 2PG/PC/PE, PC/PE, and 2TAP/PC/PE membranes, respectively. (**C**) Voltage dependences of the on-rate constant *k*_*on*_ of α-syn-VDAC binding in the membranes of different lipid compositions. The solid lines represent a fit to Boltzmann equation described in Fig. 1C with is the effective “gating charge” *n* = 3.7 ± 0.5. Data points represent the mean of at least three independent experiments ± S. D. (*error bars*).

An effect of the lipid headgroup on the α-syn-VDAC interaction is observed in 1 M KCl, where most of the lipid and protein charges are supposed to be screened (Figs 1 and 2). This result supports an earlier proposed role of hydrophobic interactions in α-syn-lipid binding^8,21,22^. To analyze the role of electrostatic contributions to the α-syn-VDAC interaction, a similar set of experiments was performed at the lower, more physiologically relevant salt concentration of 150 mM KCl (Fig. 3). The on-rate of α-syn-VDAC binding is systematically higher in 150 mM than in 1 M KCl for all three lipid compositions (Supplementary Fig. S1). Surprisingly, in low salt the on-rate appears to be rather insensitive to the presence of anionic PG in the PC/PE mixture and is ~10 times higher than that in cationic membranes (Fig. 3B,C).

These results demonstrate that on-rates of VDAC blockage by α-syn, well-resolvable at nanomolar bulk concentrations, depend on the lipid headgroup charge (anionic PG vs cationic TAP vs neutral PC/PE in 1 M KCl and PG vs TAP in 150 mM KCl), lipid packing stress (lamellar PC versus nonlamellar PE in 1 M KCl), and electrolyte screening (1 M vs 150 mM KCl). The on-rate depends on the local concentration of membrane-bound α-syn near the VDAC pore and therefore should be sensitive to α-syn membrane-binding efficiency. We expect that if the α-syn-VDAC interaction does not discriminate between different conformations of the membrane-bound α-syn molecule, then the dependence of the on-rates on lipid composition will directly correlate with efficiency of α-syn-membrane binding measured by macroscopic methods.

### α-Syn binding to the planar lipid bilayers from BOA

One such macroscopic method is BOA, in which the second harmonic of the bilayer electric response is analyzed to report on the electrical properties of both the bilayer and the membrane-bound molecules and to provide real-time kinetics of protein-membrane binding^41–44^. The attractive feature of BOA is that it allows to study synuclein binding on exactly the same system as in the VDAC nanopore experiments and uses unlabeled protein. It measures a trans-membrane potential Δ*ψ* which arises from asymmetry between the two lipid leaflets. In our system, α-syn bears net negative and slightly net positive charges on its C-terminal and N-terminal domains, respectively, and induces asymmetry in the trans-membrane potential if bound to one side of the membrane, with the sign of Δ*ψ* presumably defined by the charges bound to the membrane within the Debye length from the surface or by the modification of the lipid headgroup dipolar moment.

**Figure 4:**
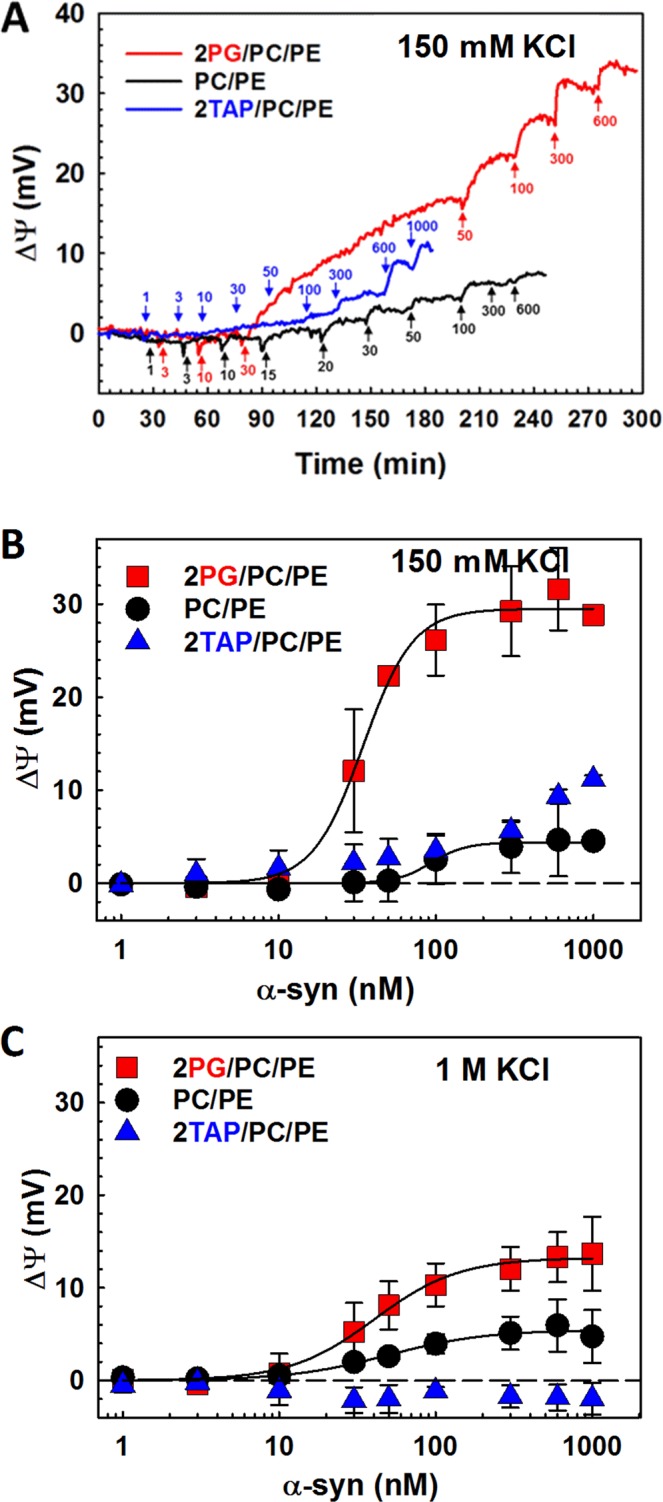
α-Syn binding to the planar lipid membranes obtained with BOA. (**A**) Representative BOA experiment reporting a time course of α-syn binding to the 2PG/PC/PE, PC/PE, and 2TAP/PC/PE membranes in 150 mM KCl, pH 7.4. α-Syn additions to the *cis* side of the membrane are shown by arrows; labels indicate the total concentration of α-syn in nM to which the bilayer was exposed until the next addition. (**B**,**C**) Curves of α-syn binding to the membranes of three lipid compositions in 150 mM (**B**) and 1 M KCl (**C**). The solid lines represent fit to the Hill equation with parameters Δ*ψ*_max_ equal to 30.6 and 5.5 mV in 150 mM KCl (**B**) and 13.2 and 5.7 mV in 1 M KCl (**C**) for 2PG/PC/PE and PC/PE membranes, respectively, and Hill coefficient of 2–2.5 for 2PG/PC/PE and 1.4 for PC/PE membranes. Data points and error bars represent the mean and S. D. for three independent experiments.

The examples of time course of trans-membrane potential changes obtained for three lipid compositions in 150 mM KCl are shown in Fig. 4A. These data provide the binding curves for 150 mM and 1 M KCl shown in Fig. 4B,C, respectively. The results demonstrate that α-syn binds more effectively to anionic membranes, with especially pronounced binding in low salt. Interestingly, even though α-syn can block VDAC in 2TAP/PC/PE membranes in 1 M KCl (Fig. 1), it does not induce a measurable trans-membrane potential in cationic membranes in high salt (Fig. 4C). In neutral PC/PE membranes, the binding curves are similar for the high and low salt conditions (Fig. 4B,C). The amount of bound α-syn in 1 M KCl, assumed to be proportional to the trans-membrane potential for all lipid compositions, follows the same sequence as in the channel experiments obtained in high salts: 2PG/PC/PE > PC/PE > 2TAP/PC/PE (Figs 1C and 4C). However, in 150 mM KCl the binding sequence deduced from macroscopic BOA measurements: 2PG/PC/PE > PC/PE ≥ 2TAP/PC/PE (Fig. 4B), does not follow the on-rate series obtained in channel experiments: 2PG/PC/PE = PC/PE > 2TAP/PC/PE (Fig. 3C). Surprisingly, in low salt, α-syn induces a positive transmembrane potential in cationic membranes, which indicates that the N-terminal domain binds to the positively charged membrane, presumably due to the hydrophobic interactions despite electrostatic repulsion. It should be noted that electrostatically-driven adsorption of the negatively charged C-terminal domain to the cationic membranes may compensate the transmembrane potential measured in BOA. In any case, macroscopic BOA measurements support our findings with the VDAC nanopore analysis by manifesting measurable binding effects at α-syn concentrations that are three orders of magnitude smaller than the characteristic concentrations reported previously, mostly from fluorescence correlation spectroscopy (FCS) measurements^6,45–48^.

## Discussion

The kinetic analysis of α-syn-induced VDAC blockage shows that its on-rate strongly depends on lipid composition, supporting the model where the α-syn’s C-terminal capture by the VDAC nanopore is preceded by N-terminal domain binding to the membrane^36^. The exponential dependence of the on-rate on transmembrane potential (Figs 1C and 3C) suggests that the capture rate is limited by a potential-dependent energy barrier comprising entropic and electrostatic components. This barrier was previously estimated to be quite large, ≥13*k*_*B*_*T*, reduced only by ≈0.13*k*_*B*_*T* per mV of applied potential^34^ and thus expected to dominate over the entire voltage range used in present experiments. The rate at which α-syn attempts to surmount this barrier is expected to be voltage-independent but proportional to the α-syn surface concentration. Thus, both the surface concentration and any effects that change the barrier to C-terminal domain insertion into the nanopore affect the on-rate.

The blockage on-rate is the highest in the anionic membranes (Figs 1 and 3) to which α-syn binds preferentially^21^. This is consistent with the BOA data (Fig. 4) and a number of previous studies performed with FCS^6,21,23,24^. The on-rate is systematically higher in 150 mM KCl than in 1 M (Figs 1C and 3C, and Supplementary Fig. S1), which supports the involvement of electrostatic forces in α-syn-membrane binding, as has been previously suggested^21^. Experiments in “non-physiological” 1 M salt solutions help to partially dissect the electrostatic component thus shedding extra light on the complex α-syn-membrane and α-syn -VDAC interactions. While *k*_*on*_ values span almost two orders of magnitude between anionic and cationic lipids (Figs 1C and 3C), the slope of their voltage dependences is essentially the same. This observation corroborates the notion that the second step of α-syn-VDAC interaction is voltage-driven capture of the negatively-charged C-terminal domain into the positively charged pore^36^.

The on-rate is also sensitive to the presence of non-lamellar PE lipid: Fig. 2 shows that in 1 M KCl *k*_*on*_ is ~10 times higher in PE than in PC. Jo and coauthors^23^ have found that PE drastically increases binding of α-syn to the acidic lipid vesicles in comparison to PC. Because PC and PE are electrically neutral, this effect presumably arises from the differences in headgroup packing density. The PC headgroup is significantly larger than that of PE (72 Å^2^ surface area per headgroup for DOPC^49^ vs 64 Å^2^ for DOPE)^50^, so that the smaller packing density of PE headgroups most likely defines a stronger α-syn binding^23^. The bulkier and more ordered PC headgroups could lead to an additional penalty for the α-syn N-terminal domain penetration, thus limiting binding.

**Figure 5:**
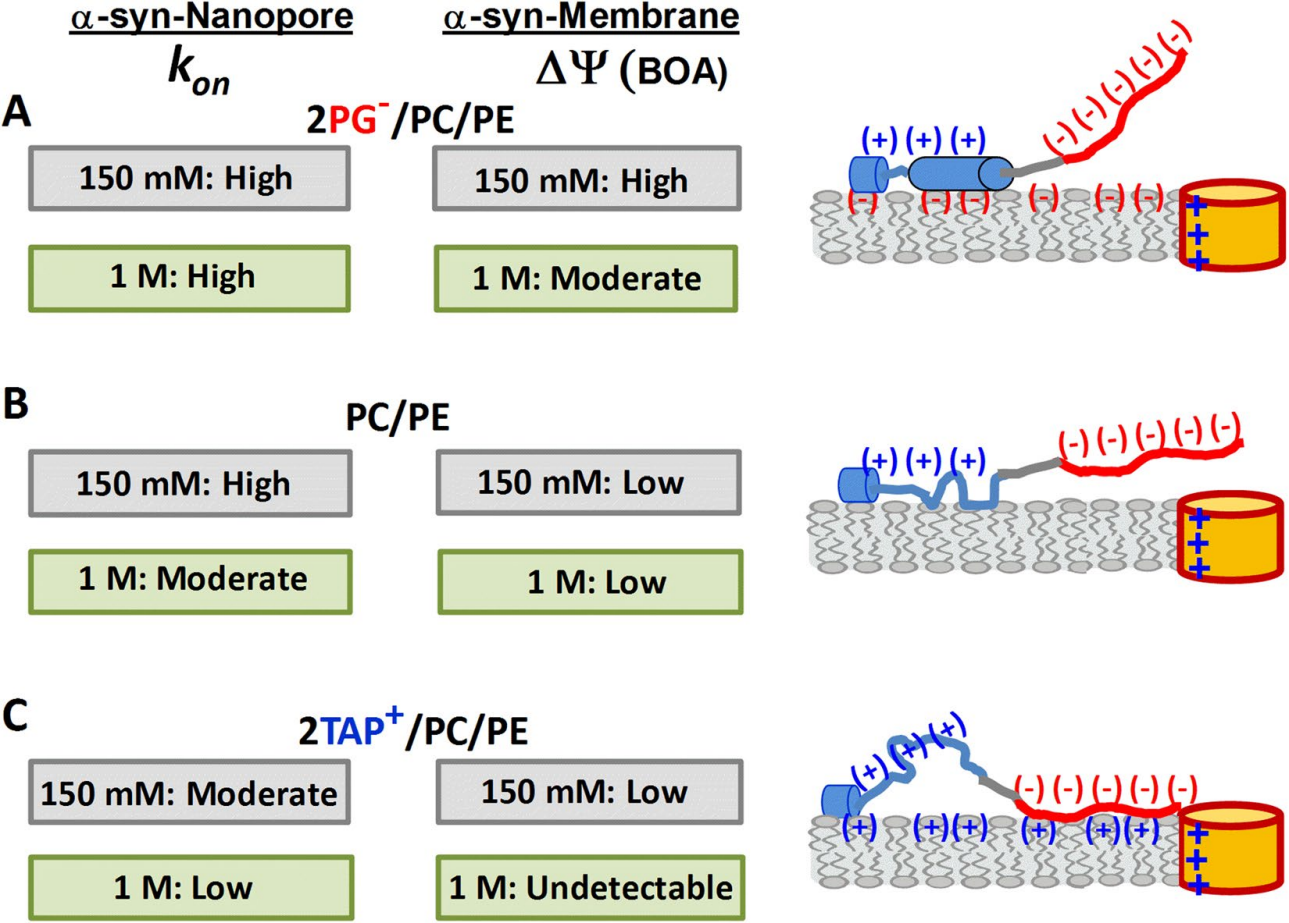
A summary of empirical findings for α-syn binding to the negatively charged (**A**), neutral (**B**), or positively charged (**C**) membranes. The cartoon on the right shows a tentative model offering a means of qualitative accounting for the electrostatic interactions and conformational variability of α-syn on the membrane surface. The availability of the anionic C-terminal domain of α-syn for VDAC pore blockage depends on the membrane lipid charge and salt concentration. The nanopore data, quantified as *k*_*on*_, the on-rate of α-syn-VDAC blockages, and α-syn-membrane binding, given as transmembrane potential Δ*ψ* measured by BOA, are presented in the left panels. Data obtained in 150 mM KCl are shown in grey boxes and those in 1 M KCl, in green. Interaction of each α-syn domain with the membrane affects the molecule’s overall membrane affinity, as well as the rate of capture of the C-terminal domain by the VDAC nanopore. The membrane-binding region is shown in blue (net 3 positive charges), nonpolar NAC domain in grey, and the anionic C-terminal domain in red (net 15 negative charges). Anionic lipids enhance α-syn binding and promote formation of two helical membrane-binding domains in the N-terminus^51,52^, but they electrostatically repel the anionic C-terminal domain from the membrane surface reducing its availability for the capture by the nanopore (**A**). In low salt, both effects are expected to be especially pronounced but, according to Supplementary Fig. S1, partially compensate each other. For zwitterionic and perhaps cationic lipids, a more random configuration of the N-terminus can be expected^22^, resulting in multiple α-syn conformations on the membrane surface (**B**,**C**), especially in high salt where electrostatic attraction is reduced. The increase in conformational variability on neutral and cationic membranes is shown as a loss of helical structure of the second part of the N-terminus domain. The effect of salt on α-syn binding to the zwitterionic lipids is negligible but *k*_*on*_ increases in low salt due to the electrostatic attraction between the positively charged pore and anionic C-terminus (**B**). For cationic membranes, electrostatic immobilization of the C-terminal domain on the membrane surface decreases the availability of the C-terminal domain for the capturing by nanopore resulting in low or moderate *k*_*on*_ (**C**).

All the changes in the on-rate of the C-terminus capture by the VDAC nanopore discussed so far can be understood by considering only the surface concentration of α-syn. One observation that does not follow this reasoning is the on-rate in PC/PE and 2TAP/PC/PE membranes, which is reversed relative to the binding efficiency measured by BOA in 150 mM KCl (Figs 3C vs 4B), but not in 1 M KCl (Figs 1C vs 4C). We speculate that while the surface concentration of α-syn on 2TAP/PC/PE membranes is larger than on PC/PE membranes, the availability of the C-terminus to the nanopore is significantly reduced. A tentative model accounting for these observations is presented in Fig. 5C: at low salt concentrations, the anionic C-terminal domain of α-syn is likely to interact with the cationic TAP headgroups, which reduces its availability for capture by the nanopore but increases the overall affinity of α-syn to the lipid surface. The lipid-dependent variety of α-syn conformations at the membrane surface is supported by an impressive number of studies^12,22,51–54^.

One of the more surprising results (compare Figs 1C and 3C and Supplementary Fig. S1) is the factor of 10 increase in the on-rate of α-syn on PC/PE membranes when the electrolyte concentration is reduced from 1 M to 150 mM. By BOA, no significant change in binding efficiency is observed between the two salt concentrations for these lipids (Fig. 4B,C), so the conclusion is that the energy barrier to insertion is lower in the 150 mM salt, presumably because of the reduction in screening of the electrostatic attraction between the acidic C-terminal domain and the positively charged pore lumen.

The on-rate of α-syn VDAC blockage for 2PG/PC/PE increases by only a factor of 2 when the salt concentration is reduced. Thus, either the binding is more efficient at high salt concentration (unlikely given the BOA results), or the availability of the C-terminal domain is much lower at low salt concentrations. The latter appears likely due to electrostatic repulsion of the anionic C-terminal domain from the anionic lipids (Fig. 5A, cartoon). Similarly, the on-rate of α-syn on 2TAP/PC/PE increases by only a factor of 4 when the salt concentration is reduced. This suggests that this could be due to the strong electrostatic attraction of the C-terminal domain to the cationic membrane surface, which makes the domain less available to the pore at low salt concentration (Fig. 5C cartoon). Thus, at the physiologically low salt concentration, α-syn binding to VDAC is less sensitive to the amount of charged lipids. Based on this observation, we speculate that *in vivo* this could be a means of self-protection against α-syn mitochondrial toxicity even as the α-syn binding to MOM goes up with the charged lipid content, such as CL, PA and PI^38^.

Most importantly, we find that at sub-micromolar concentrations, orders of magnitude below those reported as characteristic in FCS experiments, not only VDAC nanopore but BOA easily detected α-syn binding. One intriguing possibility is that the α-syn sample contains a small fraction of small oligomers that bind with higher affinity to the lipid membrane and/or oligomerization is catalyzed by the membrane surface. Dimerization on anionic lipid surfaces has been reported previously^55,56^. This also means that we cannot completely rule out a scenario in which the nanopores are probing oligomeric structures on the anionic lipid surface, rather than monomeric α-syn, and that some of the differences observed are due to differences in the structure and stability of oligomers on lipid membranes of different composition. However, in any case, our results clearly demonstrate a functionally important α-syn/VDAC interaction at its nanomolar bulk concentrations.

**Figure 6:**
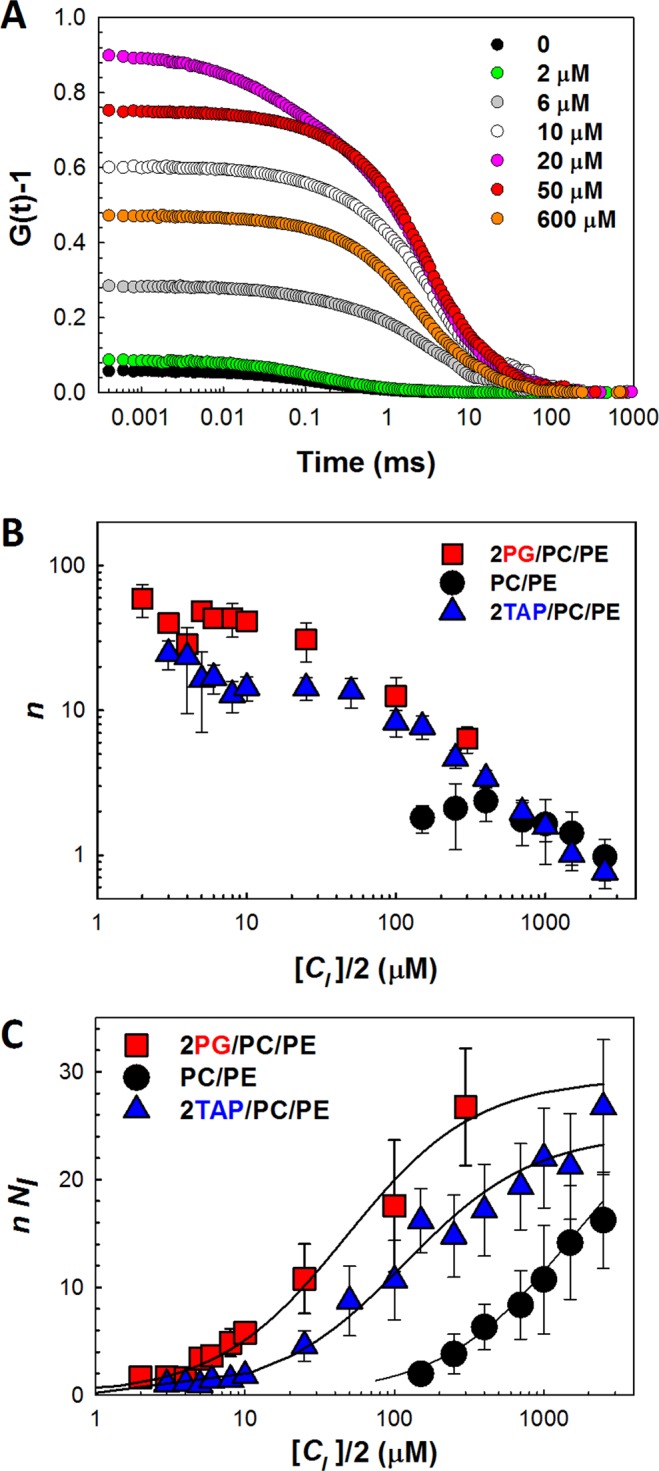
α-Syn binding to the liposome membranes measured by FCS. (**A**) Representative autocorrelation functions show how the amplitude, *G*(0) − 1, peaks as the lipid concentration is increased. The concentration of Alexa488-labeled α-syn (measured to be 30 nM) was kept constant, while the concentration of liposomes was increased. Total lipid concentrations are indicated in the panel in μM. Liposomes were formed from 2PG/PE/PC. (**B**) Average numbers of labeled α-syn molecules bound to one liposome *n*, calculated using Eq. (1), given vs. the accessible lipid concentration, [*C*_*l*_]/2. (**C**) The product *nN*_*l*_ as a function of [*C*_*l*_]/2. *N*_*l*_ is the number of liposomes in the effective illuminated volume of FCS instrument, as calculated according to Eq. (2) and the measured liposome diameter for each lipid composition. Solid lines represent fits to the simple binding curve with *K*_*d*_ equal to 47, 116, and 1500 μM for 2PG/PC/PE, 2TAP/PC/PE, and PC/PE, respectively. Maximum values of *nN*_*l*_ vary between 24–29 and are close for all lipid compositions. Liposome buffer consisted of 150 mM KCl, 5 mM HEPES at pH 7.4. Data points and error bars represent the mean ± S.D. for 3–4 independent experiments.

Finally, to reconcile our findings with the results of FCS, which reports α-syn dissociation constants in the range of several tens or even hundreds of micromolar, we performed FCS measurements with liposomes of lipid compositions used in our nanopore and BOA experiments. The FCS autocorrelation curves are shown in Fig. 6A for the increasing concentration of non-labeled 2PG/PE/PC LUVs in 150 mM KCl at a constant concentration of Alexa488-labeled α-syn of 30 nM (determined by comparison to an Alexa 488 dye standard). The shift in characteristic autocorrelation time due to the increasing fraction of liposome-bound α-syn was shown previously in number of works^6,24^. There is also a significant, non-monotonic dependence in the amplitude of the unnormalized autocorrelation function, expressed as *C*(*τ*) = *G*(*τ*) − 1, on the lipid concentration. In the limit *τ* → 0, *C*(0) reports on the inverse number of particles in the focal volume *V*, where the contribution from each fluorescent species is weighted by the square of its brightness.

Our approach to the FCS data analysis is described in Methods. We adopt two main assumptions: (i) the brightness and the total number of labelled α-syn molecules, *N*_*a*_, in the focal volume do not change during sample titration by lipid; (ii) the average number of labelled α-syn bound to one LUV is distributed according to Poisson statistics (this is not expected to be the case with strongly cooperative binding). Under these assumptions the average number of bound labelled α-syn molecules per vesicle *n* is:

1 $$n=\sqrt{\frac{1}{{N}_{l}\,{C}_{a}(0)}(\frac{C(0)}{{C}_{a}(0)}-1)}.$$

In this formula, *n*, *C*(0), and the number of lipid vesicles in the FCS focal volume *N*_*l*_ change with the lipid concentration *c*_*l*_= [*C*_*l*_]·*NA* (dimensionality 1/L), where *NA* is Avogadro's constant and [*C*_*l*_] is the preparative lipid concentration. *C*(0) and *C*_*a*_(0) are measured in the FCS experiment in lipid-containing and lipid-free samples, respectively, *N*_*l*_ is calculated from the lipid concentration and the diameter of liposomes *d* as

2 $${N}_{l}=\frac{1}{2}\frac{{c}_{l}\,V{A}_{l}}{\pi {d}^{2}},$$

where *A*_*l*_ is an area per a lipid molecule that was taken to be 0.7 nm^2^ ^57^. The focal volume *V* = 1.2 · 10^−15^ L was determined from a calibration measurement with Alexa 488 dye, while the liposome diameter *d* was directly measured by dynamic light scattering. The average number *n* of liposome-bound labeled α-syn per vesicle are calculated and plotted in Fig. 6B against total accessible lipid concentration, [*C*_*l*_]/2 (half the lipid concentration used to prepare the liposomes, assuming that α-syn binds to only the outer leaflet of liposome membrane)^6^. In the high [*C*_*l*_] limit, *n* ∝ 1/[*C*_*l*_]. This representation of FCS data is convenient for comparing and quantification of α-syn binding to different lipids and involves fewer parameters than multicomponent fitting of autocorrelation functions.

The difference in α-syn membrane binding to the different lipids is best seen if the results are presented in the form of the product *nN*_*l*_ (the number of vesicle-bound proteins in the focal volume) as a function of the same variable [*C*_*l*_]/2 (Fig. 6C). Our goal is to evaluate lipid concentration the [*C*_*l*_]* corresponding to the reduction of the unbound α-syn concentration by a factor of 2, i.e., *nN*_*l*_ = *N*_*a*_/2. This concentration gives us the dissociation constant of α-syn binding as *K*_*d*_ = [*C*_*l*_]*/2. From our data we obtain the following *K*_*d*_: 47, 116, and 1500 μM for 2PG/PC/PE, 2TAP/PC/PE, and PC/PE, respectively. These dissociation constants are in good agreement with the data for similar lipid compositions from other laboratories, thus demonstrating that the systems under study are similar, if not identical, to those studied before (e.g., refs ^6,24^).

Being a directly quantifiable method, FCS allows us to estimate the lipid surface density of α-syn. It depends on the lipid species and the ratio between its bulk concentration and that of lipids available for binding. Figure 6B shows that at 100 μM of lipids and 30 nM α-syn, the average number of synuclein molecules bound per liposome is about 2 for PC/PE, 10 for 2TAP/PC/PE, and 15 for 2PG/PC/PE. Taking into account the surface area of a 100 nm diameter liposome, we arrive to the following estimates for the average membrane area per one membrane-bound α-syn molecule: ~1.5 · 10^4^ nm^2^ for PC/PE, ~3 · 10^3^ nm^2^ for 2TAP/PC/PE, and ~2 · 10^3^ nm^2^ for 2PG/PC/PE.

To conclude, FCS does yield orders of magnitude higher dissociation constants for α-syn binding to membranes of similar lipid compositions than the characteristic concentrations of α-syn necessary for the functional blockage of VDAC described here. The nanomolar potency of α-syn was previously shown for the plant lipid diphytanoyl-PC, but now, importantly, is also demonstrated for MOM-mimicking lipids, including those used in previously reported FCS measurements.

## Conclusions

We find that functionally important α-syn binding begins at nanomolar solution concentrations, three orders of magnitude smaller than those determined by the macroscopic measurements. This finding highlights the complexity of peripheral protein interactions with membranes. Our results also suggest that bound α-syn can adopt different sub-conformations on the membrane surface depending on the lipid charge and headgroup packing density, and that each sub-conformation is characterized not only by a different membrane binding affinity, but also by a different availability of the C-terminus for capture by the VDAC nanopore.

More generally, we demonstrate that the β-barrel nanopore of VDAC can be used as a single-molecule probe of α-syn membrane binding, which reports on both the lipid-dependent concentration and conformational variability of the α-syn molecule at the membrane surface. This opens a new research avenue to the studies of peripheral proteins binding thermodynamics and kinetics of their interaction with biological membranes resolved at the single molecule level.

Our study also indicates that the functional significance of α-syn membrane binding may vary based on its solution concentration and membrane lipid composition. For the mitochondrial outer membrane mimics used in the present study, VDAC blockage by α-syn saturates in the range of nanomolar solution concentration. By contrast, this is well below concentrations that are necessary for membrane remodeling^25–27^. Such significant separation in concentration ranges may be an important feature allowing for the dual functionality of α-syn in mitochondrial regulation and in cellular membrane remodeling.

## Methods

### Materials

1,2-dioleoyl-sn-glycero-3-phosphocholine (DOPC), 1,2-dioleoyl-sn-glycero-3-phosphoethanolamine (DOPE), 1,2-dioleoyl-sn-glycero-3-phospho-(1′-rac-glycerol) (DOPG), 1,2-dioleoyl-3-trimethylammonium-propane (DOTAP) and cardiolipin from bovine heart (CL) were purchased from Avanti Polar Lipids (Alabaster, AL). All other chemicals were obtained from Sigma-Aldrich unless noted otherwise.

### Protein Purification

Recombinant mouse VDAC1 (VDAC) was a kind gift of Dr. Adam Kuszak (Laboratory of Chemical Physics, NIDDK, NIH). Protocols for expression, refolding, and purification of VDAC1 were based on the work of Dr. Jeff Abramson’s lab^33^. After size exclusion chromatography C-terminal His-tagged murine VDAC1 was finally stored in 50 mM Tris∙HCl (pH 8), 150 mM NaCl, 0.075% LDAO, 0.5 mM TCEP buffer at −80 °C. WT α-syn and its Cys variant (Y136C) were expressed, purified, and characterized as described previously^58^ and stored at −80 °C. Protein molecular weights were confirmed by ESI-MS (Biochemistry Core Facility, NHLBI). Protein concentrations were determined by an extinction coefficient of 5120 M^−1^ cm^−1^ for WT, and 4470 M^−1^ cm^−1^ for Y136C at 280 nm using a Cary 300 Bio-spectrophotometer (Varian). For labeling Y136C with Alexa Fluor 488, purified Y136C was first treated with 500 μM Tris(2-carboxyethyl) phosphine (TCEP) in 10 mM phosphate buffer for 1 hour to reduce any disulfide bonds. Then Alexa Fluor 488 C5-maleimide (ThermoFisher) was added (molar ratio of ~4:1 dye/protein) and let it react in the dark at room temperature for 2 hours with gentle stirring under Argon atmosphere. Afterwards, 1 mM DTT was added to stop the reaction. Unreacted fluorophores were removed by a HiPrep desalting 26/10 column (GE Healthcare), and then anionic-exchange chromatography (MonoQ column, GE Healthcare) was used to purify the labeled α-syn from the unlabeled. Protein molecular weight of Alexa 488-labeled protein (15099 Da) was confirmed by ESI-MS and its concentration was determined by an extinction coefficient of 73000 M^−1^ cm^−1^ at 493 nm.

### Channel Reconstitution

The mixtures of lipids were prepared from 10 mg/ml aliquots of two or three lipid solutions in chloroform, followed by drying with nitrogen and then re-dissolving them in pentane to a total lipid concentration of 5 mg/ml. Planar bilayer membranes were formed from two opposing lipid monolayers across ~70 μm aperture in the 15-μm-thick Teflon partition separating two ~1.2-mL compartments as previously described^59^. VDAC insertion was achieved by adding recombinant murine VDAC1 in 2.5% triton X-100 buffer^60^ to the aqueous phase of 1 M or 150 mM KCl buffered with 5 mM Hepes at pH 7.4 in the *cis* compartment. Potential is defined as positive when it is greater at the side of VDAC addition (*cis*). α-Syn at a final concentration of 10 nM was added symmetrically to the both sides of the membrane after VDAC channel reconstitution; statistical analysis of the blockage events was started 15 min after α-syn addition to ensure a steady state. Conductance measurements were performed as described previously^36^ using an Axopatch 200B amplifier (Axon Instruments, Inc., Foster City, CA) in the voltage clamp mode. Data were filtered by a low pass 8-pole Butterworth filter (Model 900, Frequency Devices, Inc., Haverhill, MA) at 15 kHz and a low pass Bessel filter at 10 kHz, and directly saved into computer memory with a sampling frequency of 50 kHz. For data analysis by Clampfit 10.3, a digital 8-pole Bessel low pass filter set at 5 kHz or 2 kHz was applied to current recordings in 1 M KCl and 150 mM KCl, respectively, and then individual events of current blockages were discriminated. Individual events of current blockages were discriminated and kinetic parameters were acquired by fitting single exponentials to logarithmically binned histograms^61^ as described previously^36,62^. The total number of events in the histograms was between 400 and 10,000. All lifetime histograms used 10 bins per decade. Four different logarithmic probability fits were generated using different fitting algorithms and the mean and standard deviation of the fitted time constants were used as the mean and standard deviation for the characteristic open and blockage times. Each experiment was repeated at least three times on different membranes. Records for analysis were obtained no less than 15 min after α-syn addition to ensure a steady state.

### Bilayer Overtone Analysis (BOA) measurements and data analysis

The BOA measurements were performed as described^43,44^ using a Stanford Research Systems 830 lock-in amplifier and planar lipid membranes made by the same protocol as for VDAC reconstitution experiments. An excitation potential *V*(*t*) with frequency *f*_0_ = 1933 Hz and amplitude *V*_*ac*_ = 106 mV (75 mV_rms_) and variable dc potential *V*_*dc*_ were applied to the membrane. The ac current was measured at the frequency of the second harmonic 2*f*_0_ = 3866 Hz. For a lipid bilayer membrane with a capacitance *C* that scales with applied voltage as *C* = *C*_0_ + *αV*^2^, where *C*_0_ is the capacitance in the absence of an applied potential and *α* is related to the compressibility of the membrane, the second harmonic current is^41,42^.

3 $${i}_{2}(t)=-\,6\pi {f}_{0}\alpha (\psi +{V}_{dc}){V}_{ac}^{2}\,\sin \,(4\pi {f}_{0}t).$$

Here *ψ*, the intrinsic membrane potential, reports on the asymmetry between two monolayer leaflets. Experimentally, *ψ* was determined as described^44^ from measuring *i*_2_(*t*) amplitude as a function of *V*_*dc*_, which was swept from −50 to 50 mV in steps of 10 mV; the amplitude is minimized at *V*_*dc*_ = −*ψ*. Data are represented as Δ*ψ* = *ψ* − *ψ*_*t*=0_, where positive Δ*ψ* corresponds to the increase of positive charge on the *cis* surface of the membrane. α-Syn was added stepwise to the *cis* compartment under constant stirring in this compartment. Membrane capacitance *C* and Δ*ψ* were measured approximately once per minute and stored in computer using in-house build software^44^. Each data point is an average of 10 subsequent Δ*ψ* measurements taken when the signal reached a steady-state level, as defined by Δ*ψ* variations within ±0.5 mV. It has been shown that at micromolar α-syn concentrations (or as low as 140 nM depending on the lipid composition), α-syn perforates planar lipid membranes containing acidic or nonlamellar lipids^63^. To avoid this phenomenon, our BOA measurements performed on VDAC-free planar membranes were limited to sub-micromolar concentrations.

### Fluorescent Correlation Spectroscopy (FCS) measurements and data analysis

#### Liposome preparation

Aliquots of 10 mg/ml stock lipid solutions in chloroform were mixed and chloroform was removed under nitrogen stream; the mixtures were held thereafter in a vacuum desiccator overnight. The dried lipid mixtures were re-hydrated with 150 mM KCl, 5 mM HEPES, pH 7.4 to a final concentration of 1 mM lipid for 60 min on ice with gentle agitation every 15 minutes. The lipid-buffer solutions were then vortexed for 30 seconds to fully homogenize the sample and passed through lipid extruder (Avanti Polar Lipids, Inc.) using polycarbonate membrane filters (Millipore) with the pore sizes of 200 and 100 nm, sequentially. Liposomes’ size and polydispersity were determined for each lipid composition and preparation by light scattering using Zetasizer Nano-ZS90 (Malvern). Homogeneous populations of Large Unilamellar Vesicles (LUVs) of 100 ± 26 nm diameter with a polydispersity <0.2 were used in FCS measurements. The liposome diameter measured for each lipid sample was used in analyzing the FCS data from that sample.

#### Sample preparation

FCS measurements were made in eight-well cover-glass slides (Grace Biolabs) pretreated with Sigmacoat (Sigma Aldrich) before each experiment to prevent liposome and α-syn adhesion to the surfaces. FCS measurements were carried out using a Hamamatsu Photonics K.K. C9413-01 spectrometer with a 473 nm excitation laser as described previously^64^. In order to minimize the potential influence of the fluorescent dye on α-syn-membrane binding, Alexa488 was placed in position Y136C in the C-terminus of α-syn. Considering that the N-terminal domain is generally accepted to be membrane-binding, the presence of Alexa at the C-terminus is unlikely to affect binding (see also)^6^. Samples for FCS measurements contained Alexa488-labeled α-syn alone or in liposome solution in 150 mM KCl, 5 mM Hepes, pH 7.4. The concentrations of the Alexa488-labeled α-syn were calculated relative to a calibration with free Alexa488 dye.

To interpret the results of FCS measurements we use a simple model with the following assumptions: (i) the brightness and total number of labelled α-syn molecules in the illuminated volume, *N*_*a*_, do not change upon sample titration by lipid; (ii) liposomes have the same average number of bound labelled α-syn molecules, *n*, distributed according to Poisson statistics. Here we show that this model allows a straightforward estimation of dissociation constants of α-syn binding to the membrane surfaces using only the limiting values of the autocorrelation functions at *τ* → 0. Indeed, from the first assumption it follows that all autocorrelation functions (ACs) obtained in FCS lipid titration experiments are normalized by the same 〈*I*〉^2^, where the mean fluorescence signal can be written as 〈*I*〉 = *N*_*a*_*h*, with *h* designating an “empirical brightness” of a single labelled α-syn molecule, and *N*_*a*_ standing for the mean total number of these molecules in the effective illuminated volume. Then, the AC for a sample of pure labelled α-syn molecules at *τ* = 0 can be written as

4 $${C}_{a}(0)=\frac{{N}_{a}{h}^{2}}{{({N}_{a}h)}^{2}}.$$

Addition of *N*_*l*_ liposomes reduces the number of free labelled α-syn molecules to *N*_*a*_ − *nN*_*l*_, decreasing their contribution to the AC to

5 $${C^{\prime} }_{a}(0)=\frac{({N}_{a}-n\,{N}_{l}){h}^{2}}{{({N}_{a}h)}^{2}}.$$

Simultaneously, liposome addition introduces new fluorescent particles with average brightness *nh*. Because of the second assumption, their contribution to the AC can be expressed through the second moment of the Poisson distribution, *n*^2^ + *n*, as

6 $${C^{\prime} }_{l}(0)=\frac{{N}_{l}({n}^{2}+n){h}^{2}}{{({N}_{a}h)}^{2}}.$$

The resulting AC is the sum

7 $$C(0)={C^{\prime} }_{a}(0)+{C^{\prime} }_{l}(0)=\frac{{N}_{a}+{N}_{l}\,{n}^{2}}{{{N}_{a}}^{2}}.$$

From Eq. (4) *N*_*a*_ = 1/*C*_*a*_(0); using this expression in Eq. (7), we arrive at a quadratic equation for *n*, which yields Eq. (1) of the main text.

### Statistics

For the statistical analysis of mean values, the difference between two groups of data were analyzed by a two-tailed *t*-test using *p* < 0.05 as the criterion of significance. Differences between many groups were analyzed by one-way analysis of variance.

## Supplementary information

Supplementary Information

## Data Availability

All datasets generated as part of this study are available from the corresponding author upon reasonable request.
