# Supplementary material for: Probing Membrane Association of α-Synuclein Domains with VDAC Nanopore Reveals Unexpected Binding Pattern
Source: Sci Rep. 2019 Mar 14;9:4580. doi: 10.1038/s41598-019-40979-8 (PMC6418135; doi:10.1038/s41598-019-40979-8)
Supplement: Supplementary file 1 — Supplementary Information [file 41598_2019_40979_MOESM1_ESM.pdf]

Supplementary Information

Probing Membrane Association of α-Synuclein Domains with VDAC Nanopore Reveals Unexpected Binding Pattern

Daniel Jacobs, David P. Hoogerheide, Amandine Rovini, Zhiping Jiang, Jennifer C. Lee, Tatiana K. Rostovtseva, and Sergey M. Bezrukov

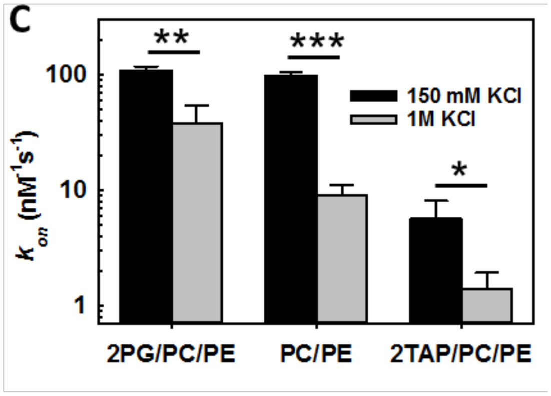

Supplementary Figure S1. The on-rate of α-Syn-VDAC binding depends on the ionic strength of membrane-bathing solution. k_{on} is greater in all tested lipid compositions (as indicated) when the membrane is bathed by 150 mM KCl in comparison with 1 M KCl. Significance was determined using a two-tailed *t*-test between 1 M and 150 mM KCl datasets for each lipid composition (***$p < 0.00003$; $^{**}p < 0.005$; $^{*}p < 0.05$;). k_{on} values are obtained at – 35 mV. Data are the mean of at least three independent experiments ± S. D. (*error bars*).
